# Synthesis of New Hybrid Structured Magnetite Crosslinked Poly Ionic Liquid for Efficient Removal of Coomassie Brilliant Blue R-250 Dye in Aqueous Medium

**DOI:** 10.3390/molecules27020441

**Published:** 2022-01-10

**Authors:** Abdelrahman O. Ezzat, Ahmed M. Tawfeek, Jothi Ramalingam Rajabathar, Hamad A. Al-Lohedan

**Affiliations:** Chemistry Department, College of Science, King Saud University, Riyadh 11451, Saudi Arabia; atawfik@ksu.edu.sa (A.M.T.); hlohedan@ksu.edu.sa (H.A.A.-L.)

**Keywords:** magnetic nanoparticles, ionic dye, water treatment, 4-vinylpyridine-co-maleicanhydride, poly ionic liquid

## Abstract

In this work, new crosslinked pyridinium poly ionic liquid and its magnetite hybrid structured composite were prepared and applied to remove the toxic dye Coomassie Brilliant Blue (CBB-R250) from aqueous solutions. In this respect, vinyl pyridine, maleic anhydride, and dibromo nonane were used to prepare crosslinked quaternized vinyl pyridinium/maleic anhydride ionic liquid (CQVP-MA). Furthermore, a linear copolymer was prepared by the reaction of vinyl pyridine with bromo nonane followed by its copolymerization with maleic anhydride in order to use it as a capping agent for magnetite nanoparticles. The monodisperse MNPs were incorporated into the crosslinked PIL (CQVP-MA) by ultrasonication to prepare CQVP-MA/Fe_3_O_4_ composite to facilitate its recovery using an external magnetic field and enhance its adsorption capacity. The chemical structures, thermal stabilities, zeta potential, particle size, EDS, and SEM of the prepared CQVP-MA and CQVP-MA/Fe_3_O_4_ were investigated. Adsorption kinetics, isotherms, and mechanisms of CB-R250 elimination from aqueous solutions using CQVP-MA and CQVP-MA/Fe_3_O_4_ were also studied, and the results revealed that the pseudo second-order kinetic model and the Langmuir isotherm model were the most suitable to describe the CBB adsorption from an aqueous solution. The adsorption capacities of CQVP-MA and CQVP-MA/Fe_3_O_4_ were found to be 1040 and 1198, respectively, which are more than those for previously reported material in the literature with reasonable stability for five cycles.

## 1. Introduction

The textile industry has globally undeniable importance as it contributes to 7% of the total world exports. In spite of the great importance of this industrial sector, the drainage of untreated effluents into water bodies causes serious environmental problems [1,2]. Dyes are considered complex, poisonous, and low biodegradable structures; hence, different techniques have been developed to purify industrial wastewater from these organic pollutants. Methods including membrane filtration, degradation, chemical oxidation, electrochemical treatment, reverse osmosis, ion exchange, and adsorption have been applied to remove pollutants from water [3,4,5,6,7]. The advantages of simplicity, high efficiency, and low cost give the adsorption method priority among other technologies [8,9,10]. Until now, various adsorbents have been proposed to remove dyes and heavy metals from wastewater such as carbon materials [11], zeolites [12], clay minerals [13], and hydrogel [14]; after the completion of the adsorption process, adsorbents are separated from the medium using filtration or centrifugation. These processes are time- or energy-consuming and may lead to loss of the adsorbent’s mechanical properties. Furthermore, some adsorbents in the nanoscale are hardly separated from the adsorption solution, which may have harmful consequences [15].

Ionic liquids (ILs) are potential environmentally friendly solvents with a melting point less than 100 °C. Recently, ionic liquids (ILs) received more attention over conventional solvents because of their low volatility, good dissolving ability, recyclability, nonflammability, high thermal and chemical stabilities, and high ionic conductivity [16]. Additionally, ILs have been frequently used for improving the adsorption efficiency of adsorbent materials. For instance, Naseeruteen et al. [17] modified the chitosan surface with 1-butyl-3-methylimidazolium ionic liquid to enhance its capacity for malachite green dye (MG) removal from aqueous solutions. Their work showed that chitosan ionic liquid beads were porous with promising adsorption capacity toward MG dye. Thangaraj et al. [18] synthesized a series of crosslinked poly ionic liquids with various counter anions and studied their capacities to remove Cr(VI) from aqueous solutions. They found that the prepared crosslinked PILs with Cl^−^ ion as a counter ion had the highest removal capacity. One of the drawbacks of using adsorbents for heavy metals and dye removal is the difficulty to completely separate the adsorbents from aqueous solutions after the removal process. In our previous work [19], amino silane and magnetite nanoparticles were used to prepare core–shell magnetite/imidazolium ILs composites, and they were applied for the removal of CBB dye from aqueous solutions. The maximum adsorption capacity of the prepared materials was 460.3 mg·g^−1^ [20].

In the current study, magnetic pyridinium crosslinked ionic liquid was synthesized by a facile method and utilized as an effective and economic adsorbent for CBB-R250 dye removal from aqueous solutions. The optimum conditions such as pH, adsorbent dosage, initial CBB concentration, and contact time were determined. The adsorption capacities of the prepared CQVP-MA and CQVP-MA/Fe_3_O_4_ reached 1040 and 1198 mg·g^−1^, respectively, to confirm that the prepared materials are excellent adsorbents for CBB dye and have great potential for practical application.

## 2. Results and Discussion

**Scheme 1:**
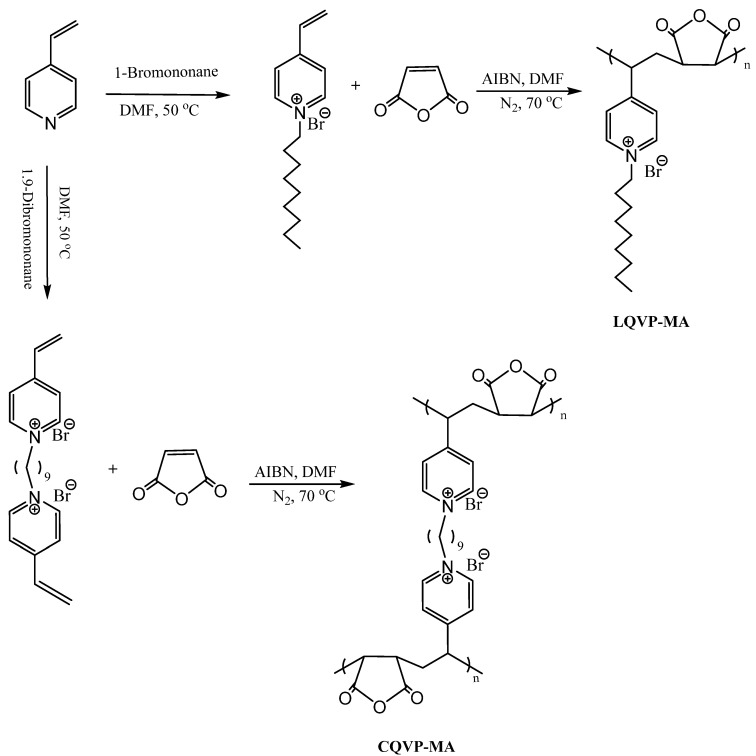
Synthesis of quaternized crosslinked and linear VP-MA copolymers.

**Scheme 2:**
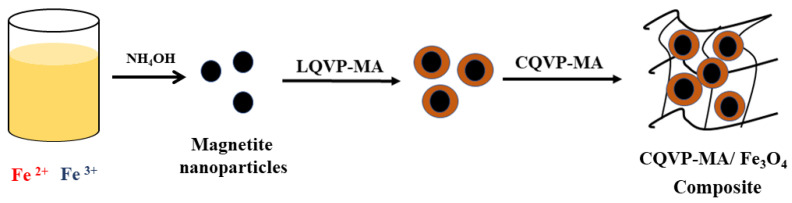
Synthesis of magnetic crosslinked PILs (CQMA-VP/Fe_3_O_4_).

Crosslinked ionic liquids have shown high removal efficiencies toward toxic dyes and heavy metals [21,22]. In this regard, new crosslinked ILs based on vinyl pyridine-maleic anhydride copolymer were prepared using dibromo alkane as a crosslinker to produce CQVP/MA. Furthermore, magnetite nanoparticles were incorporated into the network of the crosslinked IL to prepare CQVP/MA-Fe_3_O_4_ as illustrated in Scheme 1 and Scheme 2. Linear IL (LQVP/MA) was used to form monodispersed magnetite nanoparticles, as well as to form positive charges on the magnetite surface to increase its efficiency for the removal of CBB anionic dye. Furthermore, the use of magnetite in the crosslinked IL network facilitated its collection using an external magnet.

### 2.1. Characterization

**Figure 1:**
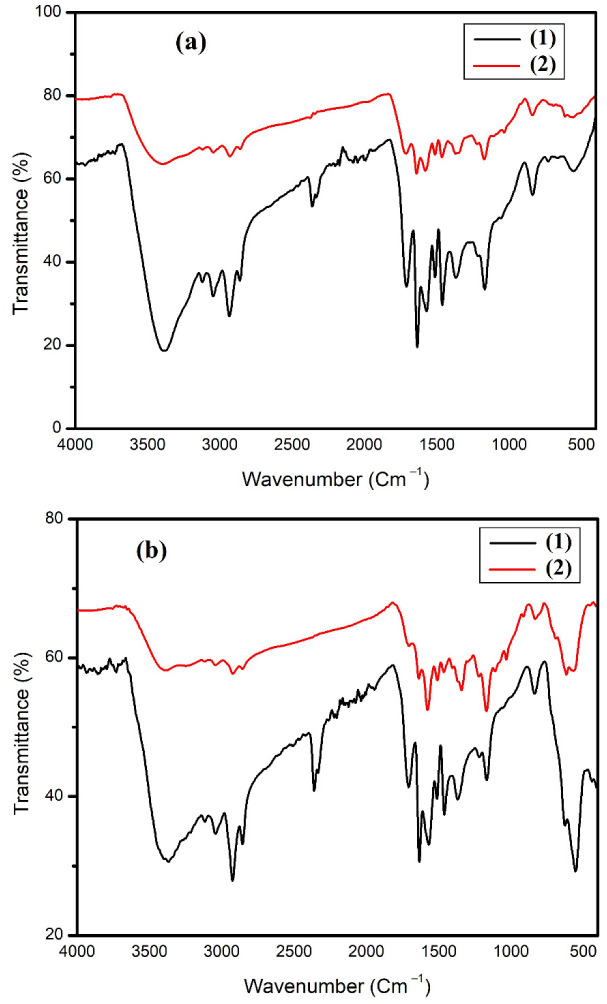
FTIR spectra of (**a**) CQVP/MA and (**b**) CQVP/MA-Fe_3_O_4_ before **(1)** and after **(2)** dye removal.

The chemical structures of CQVP/MA and CQVP/MA-Fe_3_O_4_ before and after dye treatment were confirmed using FTIR spectra as shown in Figure 1a,b. The appearance of the strong band at 540 cm^−1^ (Figure 1b) was related to the stretching vibrations of Fe–O as an indication of the Fe_3_O_4_ incorporation in the network of the crosslinked PIL (CQVP/MA-Fe_3_O_4_). The stretching vibration at 3435 cm^−1^ was attributed to the magnetite hydroxyl groups (Figure 1b). Furthermore, the appearance of strong band at 1707 cm^−1^ in CQVP/MA (Figure 1a) was due to the stretching vibration of C=O with a small shift in CQVP/MA-Fe_3_O_4_ (Figure 1b) as a result of chemisorption of magnetite nanoparticles in the polymer network. Moreover, the FTIR spectra of CQVP/MA (Figure 1a) confirm the formation of quaternary ammonium salt by the appearance of a broad peak in the range 3367 cm^−1^ [23]. Peaks at wave numbers of 3034 cm^−1^, 2925 cm^−1^, and 2857 cm^−1^ were due to the bending vibration of aromatic C–H, and stretching vibration of aliphatic asymmetric and symmetric C–H, respectively. Wave numbers of 1569 cm^−1^, 1512 cm^−1^, and 1116 cm^−1^ were assigned to C=C, C=N, and C–O stretching vibrations, respectively [24]. Additionally, the transmittance intensities of all peaks decreased as a result of CBB dye adsorption on the surfaces of the prepared adsorbents, which modified their chemical surroundings (Figure 1a,b). The variation in the chemical environment was due to the new chemical groups that are introduced to the adsorbent surfaces as a result of CBB dye adsorption [25].

**Figure 2:**
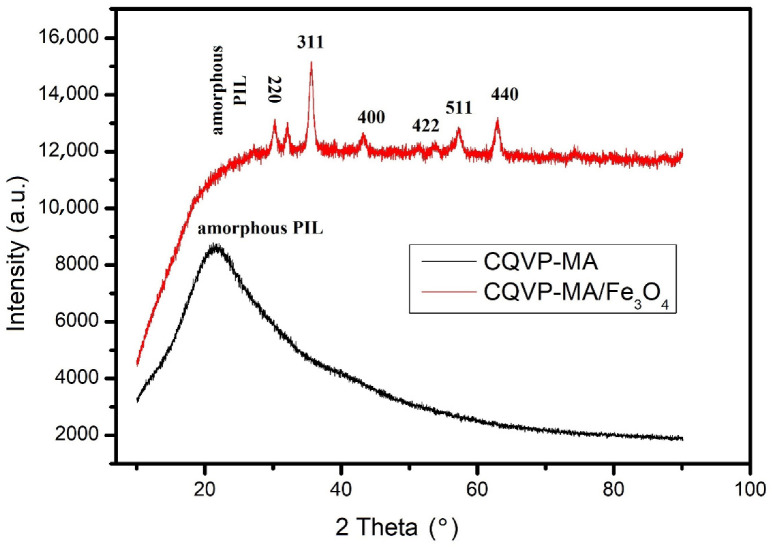
XRD diffractograms of CQVP/MA and CQVP/MA-Fe_3_O_4_.

The crystalline nature of the prepared composites was investigated using X-ray diffraction (XRD). Figure 2a,b show the XRD patterns of CQVP-MA and CQVP-MA/Fe_3_O_4_, respectively, and it is obvious that magnetite nanoparticles were quite crystalline, and the position of the diffraction peaks matched well with the standard XRD data for bulk magnetite with the formation of pure magnetite nanoparticles without other oxides [26]. The Bragg’s reflections to magnetite nanoparticles (Fe_3_O_4_ NPs) in 2θ = 30.2, 35.5, 43.4, 53.5, 57.2, and 62.8° correspond to the (220), (311), (400), (422), (511), and (440) planes, respectively. Furthermore, the formation of broad peaks for both CQVP-MA and CQVP-MA/Fe_3_O_4_ are typical for amorphous polymer networks, indicating the incorporation of magnetite nanoparticles into the polymer network [27].

**Figure 3:**
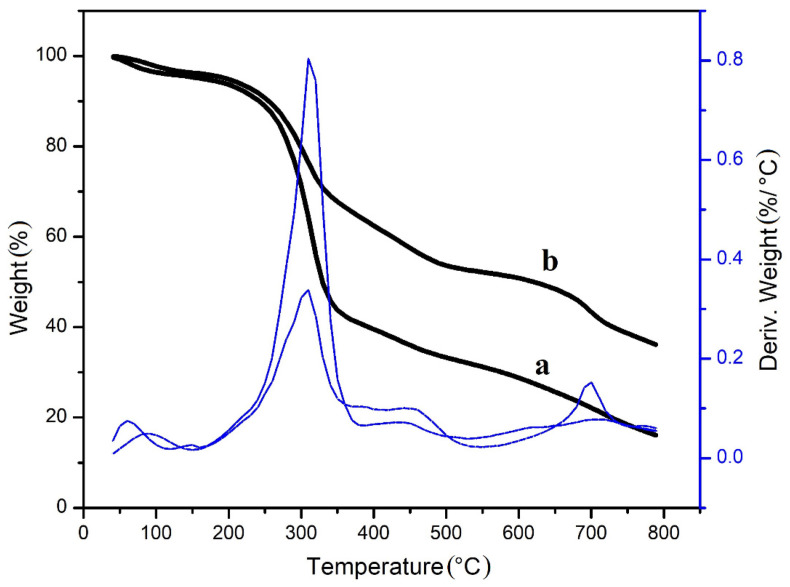
TGA thermograms of (**a**) CQVP/MA and (**b**) CQVP/MA-Fe_3_O_4_.

TGA thermograms (Figure 3) were utilized to study the thermal stabilities of the prepared crosslinked ionic liquid with its magnetite nanocomposite [28,29]. The weight losses below 200 °C in both CQVP/MA and CQVP/MA-Fe_3_O_4_ were attributed to the amount of adsorbed water molecules inside the polymer network [30], indicating the hydrophilic nature of the prepared materials due to hydroxyl groups on the magnetite surface and maleic anhydride moiety. The thermal degradation of the prepared materials started after 300 °C (Figure 3) due to the high stability of ionic liquids toward thermal decomposition [31]. The percentages of magnetite nanoparticles loaded in the IL network were calculated from the weight loss above 610 °C to be 67 wt.%. The remaining weight percentage (around 20 wt.%) in CQVP/MA at around 800 °C may have resulted from the formation of cyclic carbons [32].

**Figure 4:**
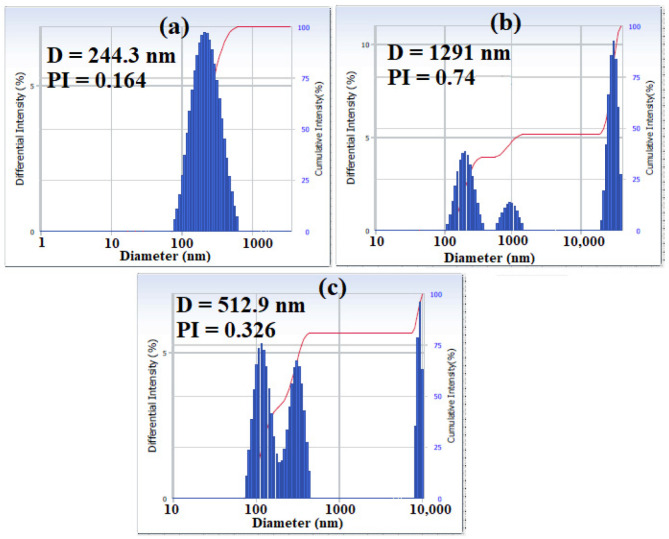
Particle size measurements of (**a**) QVP/MA-Fe_3_O_4_, (**b**) CQVP/MA, and (**c**) CQVP/MA-Fe_3_O_4_.

**Figure 5:**
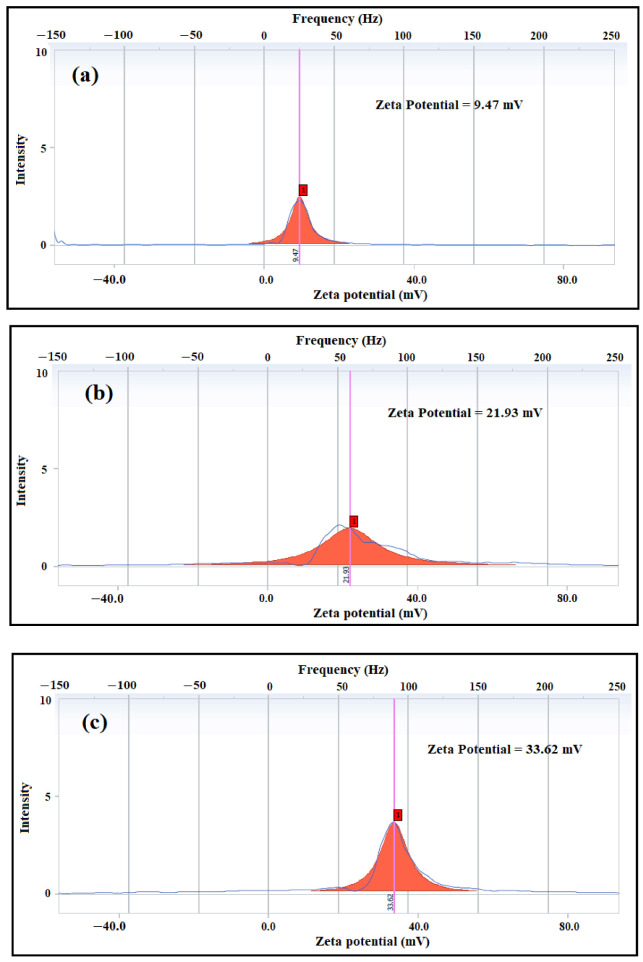
Zeta potential measurements of (**a**) QVP/MA-Fe_3_O_4_, (**b**) CQVP/MA, and (**c**) CQVP/MA-Fe_3_O_4_.

DLS was utilized to determine the particle size and surface charge of the prepared materials, as shown in Figure 4a–c and Figure 5a–c. It is indicated from Figure 4a that the linear PILs (QVP/MA) had the ability to form monodispersed magnetite nanoparticles [33] with a particle diameter of 244.4 nm and polydispersity index (PI) of 0.164. The particle size and PI of the crosslinked PIL (CQVP/MA) were 1291 nm and 0.74, respectively; these values decreased to 512 nm and 0.32, respectively, in the case of CQVP/MA-Fe_3_O_4_ because of the MNPs dispersed in the polymer network, which led to an enhancement in the dispersibility of CQVP/MA-Fe_3_O_4_. The surface charges of the synthesized materials are elucidated in Figure 5a–c, and they were 9.47, 21.93, and 33.62 mV for (a) QVP/MA-Fe_3_O_4_, (b) CQVP/MA, and (c) CQVP/MA-Fe_3_O_4_, respectively. The high positive charges on the surfaces of both CQVP/MA and CQVP/MA-Fe_3_O_4_ indicate their capability to agglutinate to anionic dyes and negative species; as a result, the prepared material can be used to remove anionic dyes and negatively charged pollutants from aqueous solutions.

**Figure 6:**
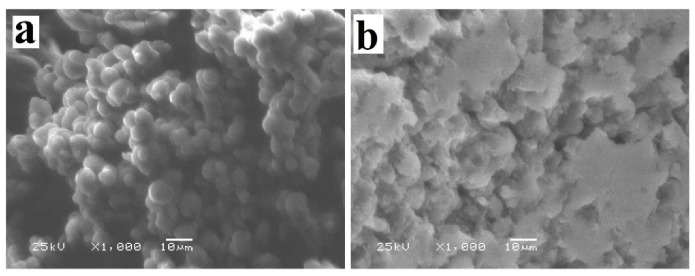
SEM micrographs of (**a**) CQVP/MA and (**b**) CQVP/MA-Fe_3_O_4_.

Scanning electron microscopy (SEM) was used to study the topography of CQVP/MA and CQVP/MA-Fe_3_O_4_, as displayed in Figure 6a,b, respectively. The SEM image of CQVP/MA (Figure 6a) shows the formation of nonuniform and stretched microspheres with a porous and extremely rough surface. Intertwining of particles led to the formation of a crosslinked framework with porous structures. As a result of MNP incorporation into the polymer cavities_,_ there was a reduction in the pore size of CQVP/MA-Fe_3_O_4_ (Figure 6b).

**Figure 7:**
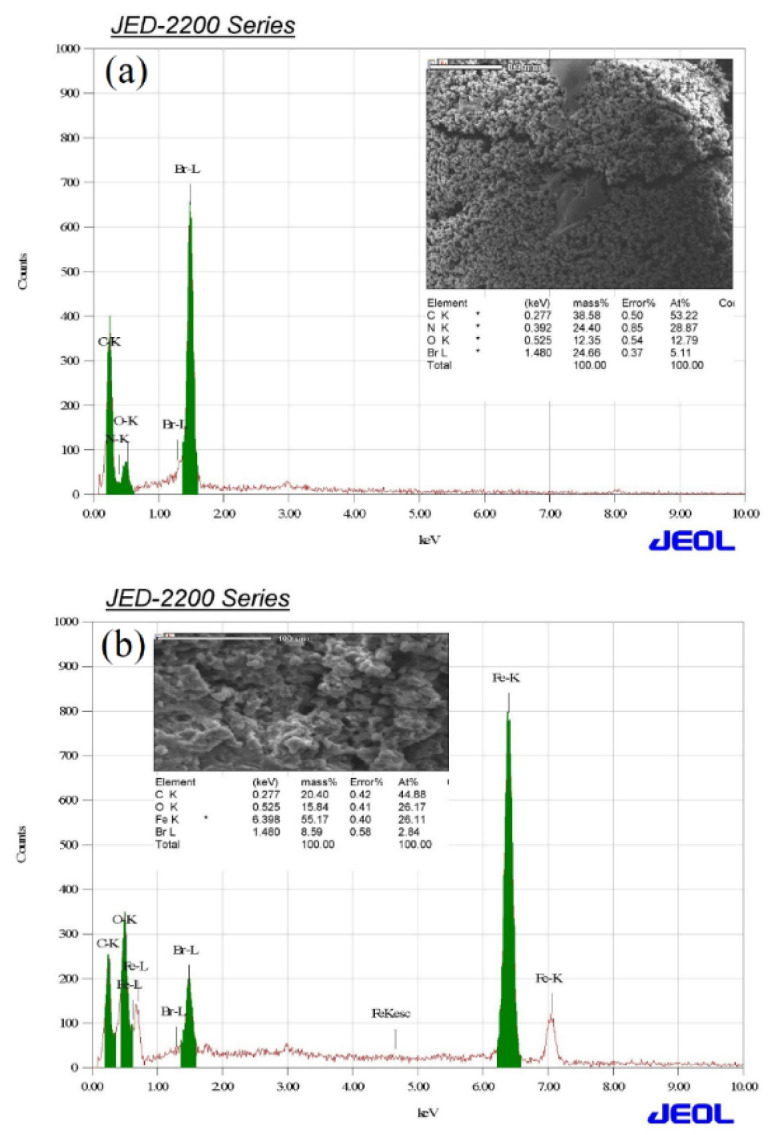
EDX results for (**a**) CQVP/MA and (**b**) CQVP/MA-Fe_3_O_4_.

The surface composition of CQVP/MA and CQVP/MA-Fe_3_O_4_ samples was determined using energy dispersion spectroscopy (EDS), as displayed in Figure 7a,b, and the results are listed in Table 1. The presence of MNPs in the PIL network was confirmed by the appearance of the Fe peak in Figure 7b, and the atomic (%) ratios of C/O/Br/Fe in CQVP/MA-Fe_3_O_4_ were 44.88/26.17/2.84/26.17, respectively (Table 1).

### 2.2. Application of CQVP/MA and CQVP/MA-Fe_3_O_4_ as CR-R250 Dye Adsorbents

Coomassie Brilliant Blue R-250 (CBB R-250) is a commercial dye used mainly in the textile industry and protein staining. In spite of its commercial value, it is considered as a toxic dye, which can lead to severe effects on human health such as cancer, shock, and heartbeat increase [34]; thus, its removal from aqueous solution is essential.

**Figure 8:**
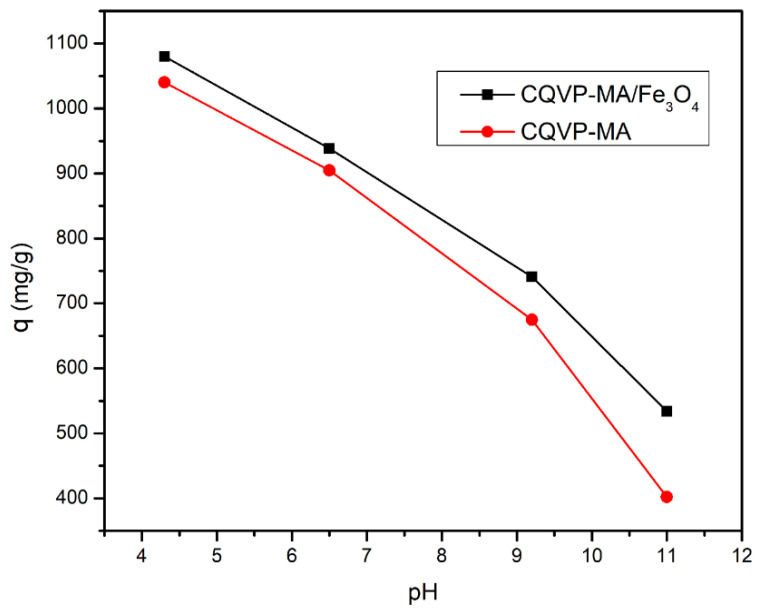
Adsorption capacity of CQVP/MA and CQVP/MA-Fe_3_O_4_ for CBB in its aqueous solution with different pH at room temperature.

The prepared crosslinked IL and its magnetic composite (CQVP-MA and CQVP-MA/Fe_3_O_4_) were applied as CBB dye adsorbents from aqueous solutions, and they were compared with previously reported materials, as shown in Table 2. To optimize the adsorption efficiencies of CQVP-MA and CQVP-MA/Fe_3_O_4_ toward the aqueous solution of CBB dye, we firstly studied the effect of solution pH on the adsorption process, as shown in Figure 8. The adsorption experiment of CBB dye was performed in the 4–11 pH range at 318 K for 12 h. It is illustrated from Figure 8 that the adsorption capacities for both CQVP-MA and CQVP-MA/Fe_3_O_4_ increased with decreasing solution pH, reaching the highest values at pH = 4. At low pH, there was a strong electrostatic attraction between the crosslinked ionic liquids with positively charged pyridinium cations and the anionic dye, thereby enhancing dye uptake [35]. At high pH, the adsorbent positive sites decreased and became negatively charged, which did not favor anionic dye uptake and led to electrostatic repulsion. Moreover, OH ions competed with dye ions and retarded the adsorption of the dye from aqueous solutions [36].

**Figure 9:**
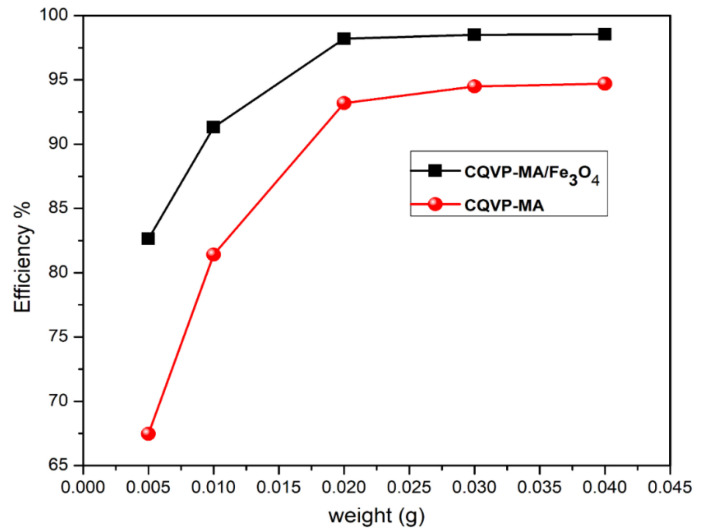
The effect of adsorbent dosage on the adsorption efficiency of CBB dye from aqueous solutions at ambient temperature.

A wide weight range of both CQVP-MA and CQVP-MA/Fe_3_O_4_ was used as adsorbents for a fixed dye volume and concentration of 15 mL and 1.8 mmol/L, respectively, to study the adsorbent dose effect on the dye removal efficiencies, as shown in Figure 9. Both CQVP-MA and CQVP-MA/Fe_3_O_4_ reached a maximum removal efficacy at an adsorbent concentration of 750 mg·L^−1^, and the removal efficiencies of CQVP-MA and CQVP-MA/Fe_3_O_4_ were 93% and 98%, respectively. The dispersed MNPs in CQVP-MA/Fe_3_O_4_ improved their adsorption performance and facilitated its collection and reusability using an external magnet.

**Figure 10:**
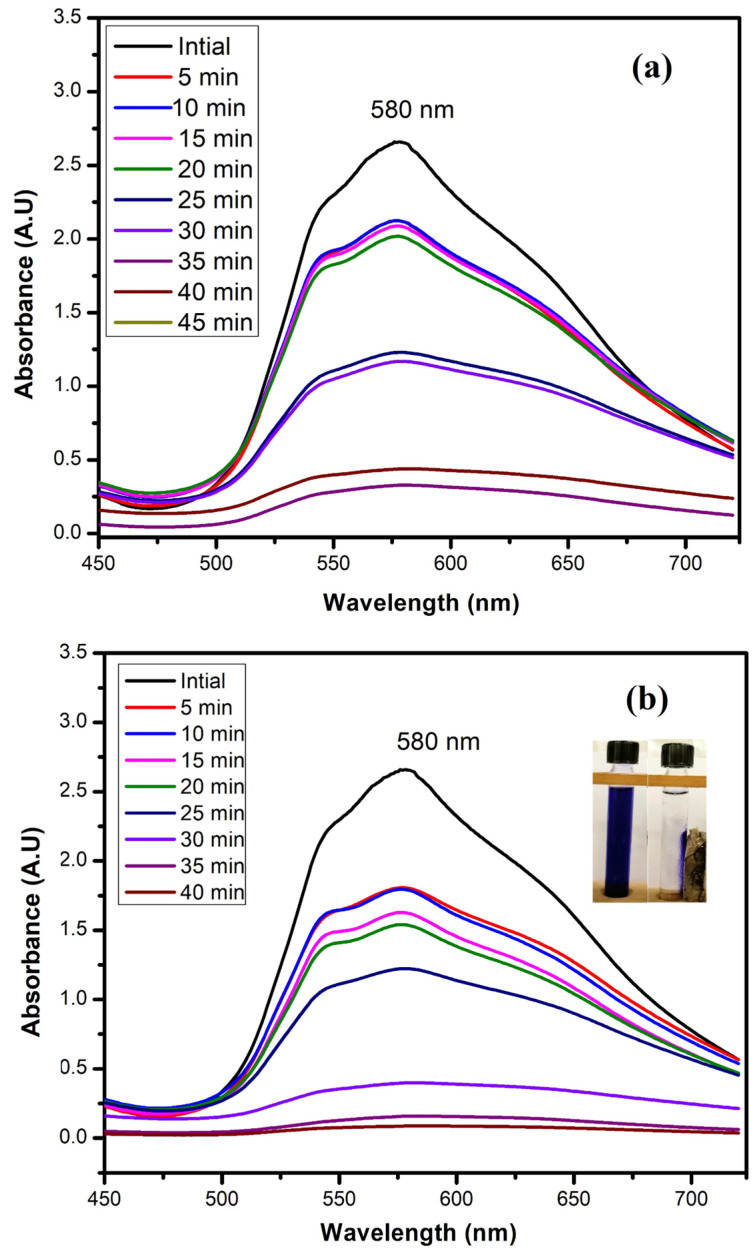
UV/Vis spectra of CBB dye solution in contact with (**a**) CQVP/MA and (**b**) CQVP/MA-Fe_3_O_4_ as a function of time.

**Figure 11:**
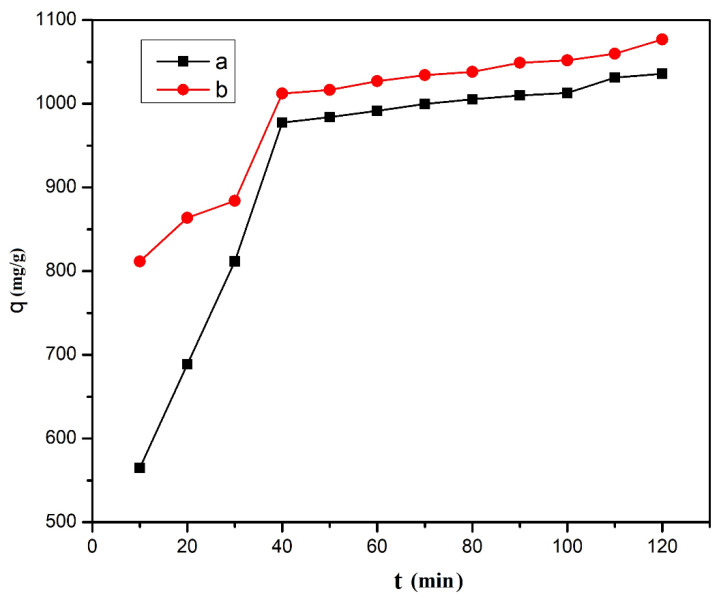
Adsorption capacity of (**a**) CQVP/MA and (**b**) CQVP/MA-Fe_3_O_4_ against time for CBB in its aqueous solution at room temperature.

The UV/Vis spectra, at λ_max_ = 580 nm, of CBB dye removal from aqueous solution using CQVP-MA and CQVP-MA/Fe_3_O_4_ adsorbents exhibited a time-dependent reduction in absorption intensity, as presented in Figure 10. The typical absorption spectrum revealed a significant removal of CBB dye in a short time with higher activity toward magnetite-incorporated adsorbent (CQVP-MA/Fe_3_O_4_) than CQVP-MA. Moreover, the adsorption capacities (mg/g) of CQVP-MA and CQVP-MA/Fe_3_O_4_ with time (min) at pH 4 and at room temperature are plotted in Figure 11. The data show that CQVP-MA and CQVP-MA/Fe_3_O_4_ reached a *q_max_* of 977 and 1012 mg/g, respectively, in a short contact time (40 min); in comparison with previous studies (Table 2), our materials had superior adsorption efficiencies toward CBB dye.

The pyridinium cations in the IL network can help in the removal of anionic dye by electrostatic interaction. Furthermore, π-π interactions between the aromatic rings in CB-R250 dye and the aromatic pyridinium rings are effective in the adhesion of dye molecules to the adsorbents [37].

### 2.3. Adsorption Kinetics and Isotherms of CBB Dye, Adsorption Mechanism of CQVP-MA and CQVP/MA-Fe_3_O_4_

The study of absorption kinetics is very useful to give information about the process of transferring dye molecules from aqueous solution to the adsorbent surface and for the adsorption mechanism. In this respect, pseudo-first-order (PFO) [38] and pseudo-second-order (PSO) [39] general kinetic models were used to investigate the adsorption of CBB onto the surfaces of CQVP-MA and CQVP-MA/Fe_3_O_4_. The PFO and PSO kinetic models are determined from Equations (1) and (2), respectively.

(1) $$\log(q_{e}-q_{t})=\log q_{e}-\frac{k_{1}t}{2.303},$$

(2) $$\frac{t}{q_{t}}=\frac{1}{k_{2}q_{e}^{2}\;}+\frac{t}{q_{e}},$$

where *q_e_* and *q_t_* (mg·g^−1^) are the CBB dye amounts adsorbed at equilibrium and time *t* (min), respectively, while *k*_1_ (min^−1^) and *k*_2_ (g·mg^−1^·min^−1^) are the rate constants in the adsorption study when applying pseudo-first-order and pseudo-second-order models, respectively. From the linear plots of PFO and PSO models, the rate constants were calculated and are tabulated in Table 3 for both CQVP-MA and CQVP-MA/Fe_3_O_4_. The calculated kinetic parameters listed in Table 3 indicate that the adsorption of CBB onto CQVP-MA and CQVP-MA/Fe_3_O_4_ is best described by the pseudo-second-order model because the correlation coefficient, *R*^2^, value was closest to 1, and the experimental adsorption capacity (*q_e_,_exp_*) values were very close to those of the calculated values (*q_e_,_cal_*).

It is important to study adsorption isotherms as they describe the interactive behavior between the CBB dye (adsorbate) and the prepared composites (adsorbents). In this respect, the adsorption isotherms were investigated using Langmuir and Freundlich models by applying mathematical Equations (3) and (4), respectively [40].

(3) $$\frac{C_{e}}{q_{e}}=\frac{1}{q_{max}K_{L}}+\frac{C_{e}}{q_{max}},$$

(4) $$\log q_{e}=\log K_{F}+\frac{1}{n}\;\log C_{e},$$

where the constants *n* (g·L^−1^), *K_L_* (L·mg^−1^) and *K_F_* ((mg·g^−1^) (L·mg^−1^)^(1/*n*)^)) are the empirical, Langmuir, and Freundlich constants, respectively, *q_e_* and *q_max_* (mg·g^−1^) are the equilibrium and maximum amount of CB adsorbate, respectively, and *Ce* (mg·L^−1^) is the CBB dye concentration in the aqueous solution at equilibrium. Table 4 shows that the experimental data for both CQVP-MA and CQVP-MA/Fe_3_O_4_ fitted better with the Langmuir model than the Freundlich model. This reveals the homogeneity of the composite surface with the formation of the CBB monolayer onto it [41].

Thermodynamic parameters, such as in the standard free energy (∆*G°*; K·J·mol^−1^), enthalpy (∆*H°*; K·J·mol^−1^), and entropy (∆*S°*; J·mol^−1^·K^−1^) were determined using the following equations [42]:

(5) $$\Delta G^{o}=-RT\;\ln K_{c},\;$$

(6) $$\Delta H^{o\;}=\Delta G^{o\;}-T\;\Delta S^{o\;},\;$$

The van’t Hoff Equation (8) was deduced from the combination of Equations (5)–(7), where *Kc* is the equilibrium constant.

(7) $$K_{c\;}=\frac{C_{ads.}}{C_{e}},$$

(8) $$\ln K_{c}=-\frac{\Delta H^{o}}{R}\;\frac{1}{T}+\frac{\Delta S^{o}}{R}.\;$$

**Figure 12:**
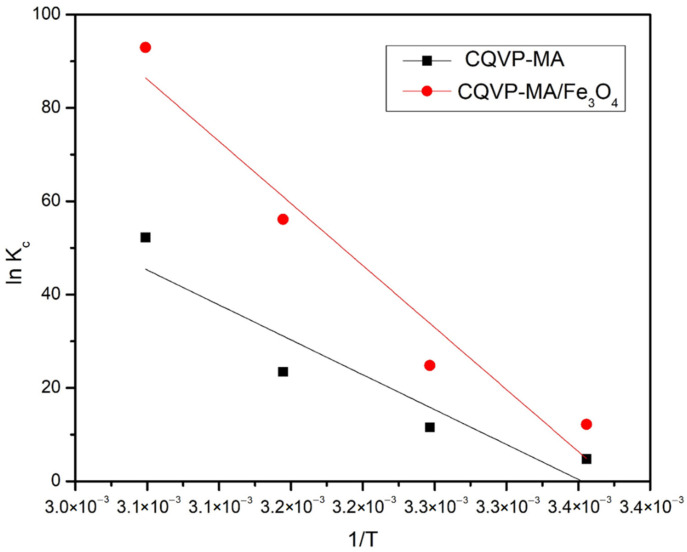
Van’t Hoff plots for the adsorption of CBB dye onto the surfaces of CQVP/MA and CQVP/MA-Fe_3_O_4_.

∆*H°* and ∆*S°* were calculated from the slope and the intercept, respectively, of the linear plot of ln *K_c_* against 1/*T* (Figure 12), and ∆*G°*, ∆*H°,* and ∆*S°* values are tabulated in Table 5. The spontaneity of the adsorption process was confirmed from the negative values of ∆*G°* at the studied temperatures. Moreover, the decrease in the value of ∆*G°* with increasing temperature suggests that the adsorption process was more spontaneous at higher temperatures. The positive values of ∆*H°* and ∆*S°* for the adsorption of CBB dye onto CQVP-MA and CQVP-MA/Fe_3_O_4_ suggest that the process was endothermic and random, respectively. The high values of ∆*H°* for both CQVP-MA and CQVP-MA/Fe_3_O_4_ reveal that there was a strong electrostatic interaction between the adsorbents and dye molecules [43].

### 2.4. Reusability Study

**Figure 13:**
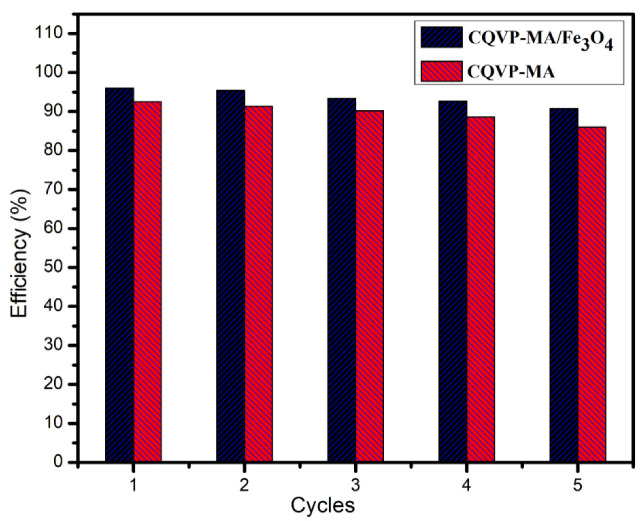
Reusability of CQVP/MA and CQVP/MA-Fe_3_O_4_ for CBB dye adsorption from aqueous solution.

The reusability of the synthesized adsorbents was tested to analyze their recapture abilities [44]. The reuse experiment was performed for five cycles using 0.02 g/L of CQVP-MA/Fe_3_O_4_ and CQVP-MA in 15 mL of aqueous solution of 1500 ppm CBB dye at pH 4. The adsorbents were regenerated after the adsorption process by stirring in acetone solvent for 2 h followed by washing with distilled water. The adsorption efficiencies for CQVP-MA/Fe_3_O_4_ and CQVP-MA displayed relatively high removal efficiencies even after five cycles, reaching 90.8% and 86%, respectively, as shown in Figure 13. Hence, the prepared adsorbents featured higher adsorption capacities over five cycles compared with the reported data in the literature, revealing their superiority for CBB-R250 removal.

## 3. Materials and Methods

### 3.1. Materials

Maleic anhydride (MA), 4-vinylpyridine (VP), 2,2′-azobis (2-methylpropionitrile) (AIBN) (BDH Chemicals, London, UK), 1-bromononane (98%), 1,9-dibromononane (97%) (Tokyo Chemical Industry Co., Tokyo, Japan), *N*,*N*-dimethyl formamide (DMF) solvent (Loba Chemie), and Coomassie Brilliant Blue R-250 (CBBr-250) (Fluka AG, chem, Buchs, Switzerland) were used without further purification. Ferric chloride hexahydrate (FeCl_3_·6H_2_O), Ferrous chloride tetrahydrate (FeCl_2_·4H_2_O), and ammonium hydroxide (NH_4_OH) were supplied by Sigma-Aldrich Co (Schaffhausen, Switzerland) and were used for the preparation of magnetite nanoparticles. Deionized water, absolute ethanol, and acetone were used as washing liquids and solvents for crosslinking polymerization. Buffer solution (H_3_PO_4_/NaH_2_PO_4_) was prepared by titration of 0.1 N of NaH_2_PO_4_ against 0.1 M HCl (for pH range 3–5) or against 0.1 N NaOH (for pH range 7–12) to adjust pH according to the required value, adjusted using the pH meter.

### 3.2. Synthesis Procedure

4-Vinyl pyridine was quaternized using an equimolar amount of nonyl bromide or half molar equivalent of dibromononane in DMF solution to prepare vinyl pyridinium bromide and divinyl dipyridinium bromide monomeric ionic liquids, respectively. The prepared monomers were copolymerized separately with maleic anhydride monomer to prepare the linear and crosslinked polymers, as shown in Scheme 1. The linear copolymer was used as a capping agent to synthesize monodisperses MNPs that were incorporated into the crosslinked PIL network to prepare the hybrid magnetic composite, as displayed in Scheme 2.

**Synthesis of linear quaternary polymer (LQVP/MA)**

First, 0.01 mol (3.12 g) of 4-vinyl pyridinium bromide (VPBr) with 0.01 mol (0.098 g) of maleic anhydride (MA) and 0.1% of AIBN initiator were mixed and dissolved in 25 mL of dry DMF solution. The mixture was heated at 70 °C with stirring for 6 h under N_2_ gas. The formed polymer was precipitated, washed with acetone, and dried in a vacuum oven at 60 °C. The yield percentage of reaction was 95% of yellow powder.

**Synthesis of crosslinked quaternary polymer (CQVP/MA)**

First, 0.01 mol (4.96 g) of 4-divinyl dipyridinium bromide (DVDPBr), 0.01 mol (0.098 g) of maleic anhydride (MA), and 0.1% of AIBN initiator were mixed and dissolved in 25 mL of dry DMF solution. The mixture was heated at 70 °C with stirring for 6 h under N_2_ gas. The formed precipitate was washed with acetone and dried in a vacuum oven at 60 °C. The yield percentage of reaction was 85% of black powder.

**Preparation of magnetite nanoparticles**

To prepare magnetite nanoparticles, ferric chloride hexahydrate (FeCl_3_·6H_2_O, 5.4 g) and ferrous chloride tetrahydrate (FeCl_2_·4H_2_O, 2.0 g) were dissolved in 100 mL of deionized water) with a molar ratio of 2:1 Fe^3+^/Fe^2+^ with stirring at room temperature. After complete solvation of ferric and ferrous salts, ammonia solution was added dropwise under continuous stirring until the reaction mixture reached pH = 10 [20]. The formed Fe_3_O_4_ NPs were collected and separated by an external magnet (made from the neodymium magnet; magnetic attraction force, 845.8 N; magnetic flux density, 534 mT), followed by sequential washing with distilled water and then drying at ambient temperature.

**Capping of magnetite nanoparticles with CQVP-MA polymer**

First, 0.5 g of magnetite nanoparticles and 0.25 g of LQVP-MA were dispersed separately in deionized water (25 mL) using a probe sonicator for 60 min and then mixed under continuous mechanical stirring for a further 12 h at 60 °C to prepare LQVP/MA-Fe_3_O_4_. The formed magnetite nanoparticles were collected using an external magnet and washed several times with ethanol and distilled water to get rid of unreacted LQVP/MA, before finally drying in a vacuum oven at 60 °C. In the same conditions, LQVP/MA-Fe_3_O_4_ (0.5 g) was mixed with CQVP/MA (0.5 g) and dispersed in deionized water (50 mL) using a probe sonicator for 60 min followed by stirring for 12 h at room temperature. The black precipitate of CQVP/MA-Fe_3_O_4_ was formed after washing several times with ethanol and dried using an oven at 60 °C. The yield percentage was 90%.

**Characterization**

Fourier-transform infrared (FTIR) spectroscopy was performed using a Shimadzu FTIR 8000 spectrometer (Kyoto, Japan) with KBr pellets. The polydispersity index (PDI) in distilled water (DW) and 0.01 M KCl was determined using a DLS (Zetasizer Nano ZS, Malvern Instrument Ltd., Malvern, UK). Scanning electron microscopy images of CQVP-MA and CQVP-MA/Fe_3_O_4_ were recorded using an SEM (Nova nano, SEM 430, FEI, Hillsboro, OR, USA). Energy-dispersive spectroscopy/energy-dispersive X-ray (EDS/EDX) elemental analysis was carried out on a Jeol SEM model JSM 6360A (Tokyo, Japan). X-ray powder diffraction (BDX-3300 diffractometer; Eindhoven, Netherlands) using a Cu anode (λ = 0.15406 nm, 30 kV, and 10 mA) was used to investigate the crystalline structure of magnetic composites at 25 °C. The thermal degradations of the synthesized composites were recorded using a TGA-50 device (SHIMADZU, Tokyo, Japan) under N_2_ flow with a heating rate of 10 °C/min. The change in the CBB dye concentration at 580 nm was determined using an ultraviolet/visible (UV/Vis) spectrophotometer (Shimadzu UV-1208 model SHIMADZU, Tokyo, Japan).

**Dye removal experiments**

A series of different CBB concentrations in aqueous solutions (5, 10, 15, 20, and 25 ppm) were prepared to establish the standard calibration curve, and the absorbance of the prepared solutions was measured using a UV/Vis spectrophotometer at a maximum wavelength of 580. The adsorption kinetics of CBB dye onto CQVP-MA and CQVP-MA/Fe_3_O_4_ was measured at different initial concentrations of CBB dye (500, 750, 1000, and 1500 ppm). To optimize the adsorption conditions, different amounts of CQVP-MA or CQVP-MA/Fe_3_O_4_ were dispersed in 15 mL of various concentrations of aqueous CBB dye solutions in a 25 mL conical flask under magnetic stirring at 25 °C. The concentration of CBB dye in the residual solution was determined using a UV/Vis spectrophotometer at wavelength 580 nm with a fixed maximum wavelength of CBB R-250 dye at different pH values ranging from 4 to 11. The amount of dye adsorption at equilibrium *q* (mg/g) and the percentage extraction (E%) were calculated from Equations (9) and (10):

(9) q=[(Co−Ce)×V/ (m)] 

(10) $$E\%=\left[\frac{\left(Co-Ce\right)\;\times \;100}{Co}\right]$$

where *Cₒ* and *Ce* (mg/L) are the liquid-phase concentrations of dye at the initial stage and at equilibrium, respectively, *V* (L) is the volume of the solution, and m (g) is the mass of adsorbent used. The same procedures were repeated at different temperatures to investigate the effect of the heat on adsorption process.

## 4. Conclusions

In the present manuscript, new hybrid materials based on crosslinked poly ionic liquids were prepared by the free radical copolymerization of divinyl dipyridinium bromide and maleic anhydride monomers. MNPs, coated with the linear copolymer (QVP-MA), were allowed to interact with the crosslinked PIL using ultra sonication to prepare a magnetic composite. The formation of the PIL network was validated by FTIR, TGA, SEM, and EDS analyses. The FTIR spectroscopy elucidated the chemical linking of magnetite NPs to the polymer network by the appearance of a peak at 626 cm^−1^ due to the Fe–O characteristic band. Furthermore, SEM images showed the formation of porous crosslinked PIL (CQVP-MA) with a pore size reduction in CQVP-MA/Fe_3_O_4_ as a result of the presence of Fe_3_O_4_ nanoparticles inside the network cavities. The porosity of the prepared materials and the positive charges of the particles as confirmed by zeta potential values highlighted their potential application as adsorbents for CBB anionic dye from aqueous solutions. The adsorption process was optimized using different parameters such as adsorbent dose and pH, and it was found to be highly dependent on the solution pH with a maximum adsorption capacity in acidic dye solutions (pH = 4). The adsorption kinetics and isotherm studies indicated that the adsorption processes of CBB best fitted the pseudo-second-order and Langmuir models, respectively. Electrostatic and π-π interactions were suggested as mechanisms to control the CBB adsorption. The adsorption capacities of CQVP-MA and CQVP-MA/Fe_3_O_4_ were 1040 and 1198, respectively, which are more than those previously reported in the literature, along with reasonable stability over five cycles, indicating that our materials are promising as inexpensive and highly effective purification systems for wastewater.

## Figures and Tables

**Table 1:** EDX quantification of elements normalized for CQVP/MA and CQVP/MA-Fe_3_O_4_.

Element	(wt.%)		(at.%)	
	CQVP-MA	CQVP-MA/Fe_3_O_4_	CQVP-MA	CQVP-MA/Fe_3_O_4_
C	38.58	20.4	53.22	44.88
O	12.35	15.84	12.79	26.17
Br	24.66	8.54	5.11	2.84
Fe	0	55.17	0	26.17

**Table 2:** Comparison of the adsorption capacities of various adsorbents for CBB at 25 °C.

System	*q_max_* mg·g^−1^	Equilibrium Time (min)	Reference
IL-functionalized ZnO NPs	53.7	90	[30]
Zn/BTMF	59.9	90	[31]
(PNCoFe) composite-120	46.06	50	[32]
Polyarginine iron oxide NPs	67.6		[33]
Acidified clays	22.89		[34]
Fe_2_O_4_-Si-IIL	460.3	275	[35]
SiO_2_-1-IIL-Fe_2_O_4_	306.9	275	[35]
CQVP-MA Fe_3_O_4_	1198	40	Current work
CQVP-MA	1040	40	Current work

**Table 3:** Kinetic parameters of pseudo-first- and pseudo-second-order models for the adsorption of CBB dye onto CQVP/MA and CQVP/MA-Fe_3_O_4_.

	*q_e,exp_* (mg·g^−1^)	Pseudo-First-Order			Pseudo-Second-Order		
		*k*_1_ (min^−1^)	*R* ^2^	*q_e,cal_* (mg·g^−1^)	*k*_2_ (g·mg^−1^·min^−1^)	*R* ^2^	*q_e,cal_* (mg·g^−1^)
CQVP-MA	1040	0.00667	0.717	691	0.000096	0.99	1111
CQVP-MA/Fe_3_O_4_	1198	0.0050	0.807	537	0.00017	0.99	1111

**Table 4:** Langmuir and Freundlich isotherms for the adsorption of CBB dye onto CQVP/MA and CQVP/MA-Fe_3_O_4_.

	Langmuir			Freundlich		
	*q_max_* (mg·g^−1^)	*K_L_* (min^−1^)	*R* ^2^	1/*n*	*K_F_* (mg·g^−1^)	*R* ^2^
CQVP-MA	1111	0.0923	0.992	0.427	154.88	0.822
CQVP-MA/Fe_3_O_4_	1250	0.066	0.963	0.545	131.83	0.949

**Table 5:** Thermodynamic parameters for adsorption of CBB dye onto CQVP/MA and CQVP/MA-Fe_3_O_4_.

	Δ*H^°^* (K·J·mol^−1^)	Δ*S^°^* (J·mol^−1^·K^−1^)	Δ*G^°^* (K·J·mol^−1^)			
			298 K	308 K	318 K	328 K
CQVP-MA	26.65	108.10	−5.83	−6.91	−8.00	−9.10
CQVP-MA/Fe_3_O_4_	27.67	123.27	−9.043	−10.28	−11.51	−12.74

## Data Availability

The data presented in this study are available on request from the corresponding author.

## Sample Availability

Samples of the materials are available from the authors.
